# Artificial solid electrolyte interphases by atomic and molecular layer deposition

**DOI:** 10.1039/d5dt00526d

**Published:** 2025-05-26

**Authors:** Milad Madadi, Ville Miikkulainen, Maarit Karppinen

**Affiliations:** a Department of Chemistry and Materials Science, Aalto University FI-00076 Espoo Finland maarit.karppinen@aalto.fi

## Abstract

Atomic layer deposition (ALD) and molecular layer deposition (MLD) are techniques known for their unique capability to produce pinhole-free and conformal thin films uniformly, even on complex 3D architectures and powders, with sub-nm thickness control. Owing to these characteristics, they are recognized as highly promising techniques for the fabrication of ultrathin protective coatings on Li-ion battery components to improve battery performance and lifetime. In the early studies, the focus was on archetypal ALD materials such as Al_2_O_3_, but recently the scope has considerably widened to cover various Li-based materials, aiming at a better ionic conductivity and enhanced Li-ion kinetics in the coating, as well as ALD/MLD-grown metal–organics with enhanced elasticity and mechanical flexibility to better moderate the volume changes in the coated electrode materials during battery charge–discharge cycling. Also, to most closely mimic the solid-electrolyte interphase (SEI) layers that naturally form in state-of-the-art Li-ion batteries, the layer should include carbonate species. In this review, we present a brief account of the current state of this exciting and timely research field, and discuss the foreseen prospects and challenges for progress.

## Abstract 2

Atomic layer deposition (ALD) and molecular layer deposition (MLD) are techniques known for their unique ability to produce pinhole-free and conformal thin films uniformly, even on complex 3D architectures and powders, with sub-nm thickness control.

## Graphical abstract

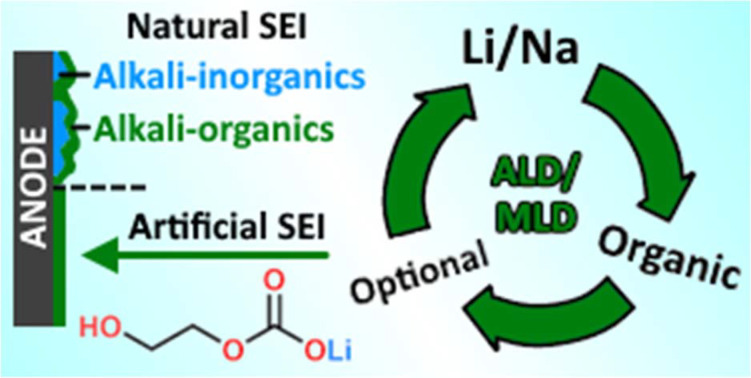

## Introduction

The rapid advancement of information technology and the unprecedented importance of clean, yet often intermittent, energy sources have led to a growing demand for affordable and reliable high-capacity energy storage. For decades, much of this demand has been met by rechargeable lithium-ion batteries (LIBs),^1^ and there have been sustained efforts to expand the technology to more abundant alkali metals, especially sodium.^2^ When these state-of-the-art alkali-ion batteries are charged, some of their liquid electrolyte decomposes onto the electrode surfaces, consuming alkali metal ions in the process and irreversibly forming a passivating and partially ion-blocking layer: a solid-electrolyte interphase (SEI).^3^ Controlling the structure of the SEI can help improve battery lifetime and even enable the use of novel, high-capacity electrode materials that deteriorate too rapidly with a spontaneously forming SEI.^4^ One way of controlling the electrode surface and the SEI formation is to coat the electrode with an engineered thin film with tailored chemical and ion-conducting properties as the protective barrier.^5^ Where such a layer particularly mimics the chemical properties of the natural SEI, it can be referred to as an artificial SEI (ASEI).

Several techniques exist for depositing ASEI layers and other electrode coatings. In this perspectival review, we focus particularly on ASEIs and related protective coatings fabricated using atomic layer deposition (ALD) and molecular layer deposition (MLD)^6,7^ techniques. These techniques enable uniform, pinhole-free and conformal thin-film coating of the electrodes, with precise control over the thickness and chemistry of the films.^6,8–10^ Thin-film conformality is vital, as any pinholes or defects in the coating structure would expose the electrode to natural SEI formation and possibly degradation.^4^ Additionally, the chemical structure of the film can be tailored to optimize ionic conductivity, chemical and mechanical stability, and any other properties desired of an electrode coating.^11–13^

## SEI layer characteristics

**Fig. 1:**
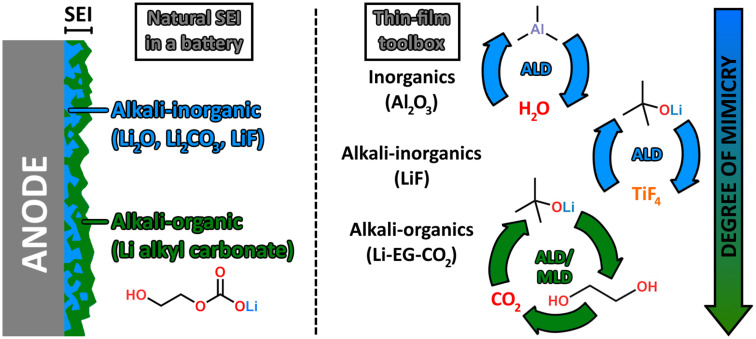
Left: A naturally formed SEI on an electrode (anode) surface, and its chemical constituents. Right: Examples of ALD and MLD coating types used to modify the anode–electrolyte interface; the degree of mimicry – *i.e.*, how well the coating chemistry mimics the chemical composition of natural SEI – increases from top to bottom.

Ever since the SEI-formation phenomenon was discovered,^14^ it has been at the heart of active research aiming to improve the performance of alkali-ion batteries – particularly the most popular among them, LIBs.^15–18^ The SEI layer conventionally results from chemical and electrochemical reactions between the negative electrode (anode) and the liquid electrolyte of a battery – though a material-dependent passivating layer does also form on the positive electrode (cathode electrolyte interphase, CEI).^19,20^ In a typical LIB, the liquid electrolyte consists of lithium hexafluorophosphate (LiPF_6_) dissolved in organic carbonates such as ethylene, dimethyl and diethyl carbonates,^16,21^ while the electrochemically active material in the anode is graphite, or sometimes an alternative material such as lithium titanate (Li_4_Ti_5_O_12_). This active material is blended with carbon black and poly(vinylidene fluoride) (PVDF) to increase electrical conductivity and to bind the materials to the current collector, respectively. As the battery is charged and discharged, the thermodynamic instability of the electrode–electrolyte interface causes the electrolyte to decompose and deposit onto the electrode surface as a SEI layer. This layer consists of inorganic lithium salts (such as LiF, Li_2_O and Li_2_CO_3_) and organic ones (ROLi, ROCO_2_Li, RCOOLi; R = organic moiety),^16,22^ with the former more concentrated in a denser inner layer and the latter in a more porous outer layer (Fig. 1) due to them forming on the electrode surface in that order.^4^ When the SEI is sufficiently thick to prevent electron conduction (at a given charging voltage and operating temperature), it prevents further reactions at the interface and stabilizes it.^18^ However, SEI formation consumes Li ions and thus depletes the storage capacity of the battery. It also increases internal resistance, decreasing the rate capability of the battery.^3,16^

Furthermore, the SEI can be inhomogeneous and defective, rather than uniform, and constantly evolving in structure in use.^23,24^ It is subject to stresses when the underlying electrode material expands and contracts upon charge–discharge cycling. Cracks appear in the SEI, resulting in uneven current and ion fluxes, and consequently even plating and stripping Li on the electrode. The newly exposed electrode surface then reacts with the electrolyte all over again and consumes more Li ions, further deteriorating the battery.^3^

**Fig. 2:**
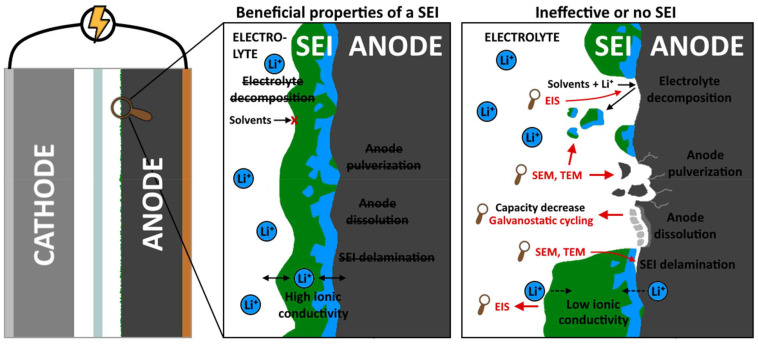
Benefits and desired properties of a properly formed SEI on an electrode, and the problems that occur without a stable SEI.

Thus, there is a clear incentive to engineer an artificial SEI layer that is chemically, electrochemically, and mechanically stable (Fig. 2), even on electrode materials that exhibit large volume changes. Various chemical and physical methods have been employed for the deposition of these ASEIs.^16,18,25–27^ Of these, ALD – particularly in combination with MLD – could provide an avenue for tailoring thin surface layers that behave optimally with the electrode material. This is enabled by the wide selection of inorganic and organic constituents, and precise control over them as they are deposited evenly, atomic and molecular layer by layer.^8,9,28,29^ Presented in this review is an overview of interesting examples of the current state of ALD- and MLD-based electrode coatings. To complement existing reviews on the subject,^12,13,30^ this work seeks to give an updated look with a particular focus on ASEIs and the negative electrode (anode) side.

## ALD and MLD process considerations

Compared to other thin-film deposition methods, ALD offers unparalleled film conformality, uniformity and control over the film structure. In ALD, gaseous precursors – the source chemicals for the materials to be deposited – are fed onto the substrate, where they chemically react through well-controlled, saturative gas-to-surface reactions. Between the precursor pulses, inert gas is used to purge (clear) the excess precursor from the gas phase. This is the key feature of ALD: the precursors react only with the surface, not amongst themselves nor with each other. In a typical ALD process, the sequence of “*first precursor pulse* – *purge* – *second precursor pulse* – *purge*” is repeated cyclically, and a controlled layer of the material is formed on the surface over each cycle. These cycles are repeated until a film of desired thickness is obtained,^29^ which is tracked by a so-called growth-per-cycle (GPC) value.^31^ The gas-diffusion-driven and surface-controlled growth in ALD yields surface coverage with atomically thin layers, and it is possible to acquire highly uniform, conformal and pinhole-free thin films,^32,33^ uniquely even on powders^13^ and challenging 3D surfaces.^34^

Most commonly, ALD processes are binary processes of a metal precursor and a second reactant, *e.g.*, trimethylaluminum (TMA, AlMe_3_) and water (H_2_O) to form aluminum oxide (Al_2_O_3_). These processes have been applied broadly to coat electrodes aimed not only at conventional batteries but also supercapacitors.^35,36^ However, more advanced materials have been made *via* ternary or quaternary processes that include three or four precursors, respectively.^37^ When organic molecules are included as precursors in combination with inorganic metal precursors, the technique is referred to as atomic/molecular layer deposition (ALD/MLD).^38^ One common ALD/MLD material is Al–ethylene-glycol (Al–EG), though the list of possible combinations is expansive.^39–41^

**Fig. 3:**
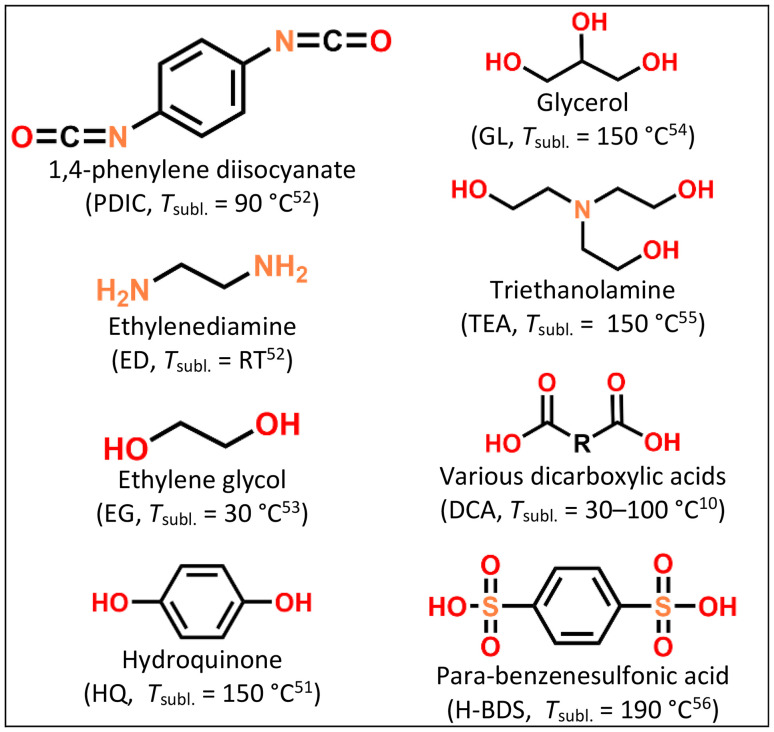
MLD (organic) precursors used for depositing thin films aimed to be used as protective or ASEI coatings on electrode surfaces, and typical sublimation temperatures used for them, and their chemical structures.

There are already a number of established ALD (Table 1) and MLD (Fig. 3) precursors used for ASEI and protective/barrier coatings for electrodes. For the ALD precursors, the list includes several alkali-metal precursors, mostly for Li. While analogues to established Li precursors exist for other alkali metals too, they often exhibit more problematic sublimation behavior and thus cannot be used as precursors. The precursors known to work are: MO^*t*^Bu (M = Li, Na, K, Rb, Cs); M–THD (M = Li, Na, K); M–TMSO (M = Li, Na) and Li–HMDS.^42^ These precursors are various degrees of air sensitive and especially moisture sensitive; this can complicate industrial application, as the ALD equipment should be designed to facilitate the insertion of solid precursors with minimal exposure to ambient air (as is already widely possible for commonly used liquid and gaseous precursors, such as the pyrophoric TMA). The resulting films may be moisture sensitive too, depending on the moiety that the alkali metal is bonded to; we suspect this to be correlated with how susceptible the bond of alkali metal and its nearest element (usually Li–O) in the material is to unwanted reactions. For example, depositions of Li with diols such as ethylene glycol and hydroquinone have yielded air-sensitive films, while Li–carboxylic-acid films tolerate ambient humidity better. The latter result in larger coordination spheres, while the former are undercoordinated, with more room for ambient water to attack and cleave the Li–O bond.^57^

**Table 1:** ALD (inorganic) precursors used for depositing thin films aimed as protective or ASEI coatings on electrode surfaces, and typical sublimation temperatures used for them (RT was assumed for the liquid precursors if not specified)

Precursor	Acronym	*T*_subl._ (°C)
Li-*tert*-butoxide, LiO^*t*^Bu	LiO^*t*^Bu	140^43^
Li-bis(trimethylsilyl)amide, LiN(SiMe_3_)_2_	Li–HMDS	55^42^
Li-2,2,6,6-tetramethyl-3,5-heptanedionate	Li–THD	175^44^
Li–trimethylsilanolate, LiOSiMe_3_	Li–TMSO	165^42^
Na-*tert*-butoxide, NaO^*t*^Bu	NaO^*t*^Bu	140^42^
Trimethylaluminum, AlMe_3_	TMA	RT^45^
Diethylzinc, ZnEt_2_	DEZ	20^46^
Titanium(iv) isopropoxide, Ti(O*i*Pr)_4_	TTIP	80^47^
Tetrakis(dimethylamino)titanium(iv)	TDMAT	45^10^
Ti–tetrachloride, TiCl_4_	TiCl_4_	RT^48^
Ti–tetrafluoride, TiF_4_	TiF_4_	120^49^
Hexafluoroacetylacetone	HFAC(H)	25^49^
Trimethyl phosphite, PO(OMe)_3_	TMP	75^47^
Tris(dimethylamino)phospine, P(NMe_2_)_3_	TDMAP	30^50^
Diethyl phosphoramidate, PO(NH_2_)(OEt)_2_	DEPA	85^51^

The as-deposited ALD and ALD/MLD films are amorphous or crystalline, depending on the material, precursor chemistry, substrate and deposition temperature.^56–61^ An amorphous structure is believed to be beneficial for solid electrolytes, to promote a higher ionic conductivity and lower electronic conductivity.^62^ The grain boundaries that commonly occur in crystalline materials can result in hampered ionic conductivity through them,^63,64^ as well as decreased material longevity due to acting as chemical and mechanical weak-points.^27^ Also, amorphous materials typically possess lower electrical conductivities compared to the crystalline ones, which is an advantage for a solid electrolyte. Finally, ALD/MLD allows for the introduction of flexible organic moieties into the structure, which is another key benefit for this particular use case, since this flexibility can aid in retaining interfacial contact between battery materials that undergo volume changes.^65^

## Substrates and characterization

**Fig. 4:**
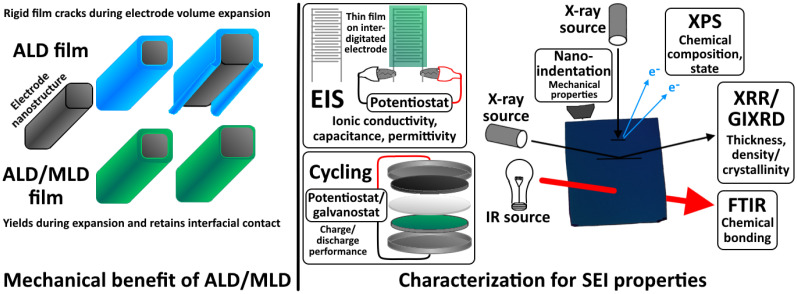
Left: Benefits to mechanical stability with electrode volume changes when depositing more flexible hybrid ALD/MLD films instead of inorganic ALD films. Right: A schematic of various techniques used to characterize thin-film electrode coatings and their properties as SEIs.

The ALD and ALD/MLD process optimization is typically performed by depositing films onto a standard flat surface, such as a silicon wafer, and varying the experimental parameters: temperatures (of the precursor and substrate), precursor pulse/purge lengths and the number of deposition cycles. Using a flat substrate allows for measuring the film thickness at a high precision with efficient techniques (Fig. 4), such as spectroscopic ellipsometry and X-ray reflectivity (XRR). It is then determinable which parameter values result in saturated, optimal film growth.^31^ This is an important process development step for ALD and ALD/MLD of ASEIs, as the electrodes – the intended end-use substrates – might not provide smooth or even sufficiently stable surfaces for process optimization purposes, thus requiring initial work using Si wafers or similar substrates. Regardless, microscopy methods have been used to evaluate film thickness for coatings deposited on electrodes.^30^

Various methods have been employed for the characterization of ALD and ALD/MLD battery material coatings (Fig. 4). These characterization methods include: atomic force microscopy (AFM), chronoamperometry (CA), chronopotentiometry (CP), synchrotron-based X-ray computed tomography (CT), cyclic voltammetry (CV), galvanostatic cycling (in test battery cells; here “Cyc”), elastic backscattering (EBS), electrochemical active surface determination (ECSA), energy dispersive X-ray spectroscopy (EDS), energy dispersive X-ray mapping (EDX), electrochemical impedance spectroscopy (EIS), focused ion beam (FIB), galvanostatic intermittent titration (GITT), inductively coupled plasma (ICP) etching, mass spectrometry (MS), nanoindentation (here “NI”), quartz crystal microbalance (QCM), Rutherford backscattering spectrometry (RBS), spectroscopic ellipsometry (SE), field-emission scanning electron microscopy (FE-SEM), bright-field/high-resolution/scanning transmission electron microscopy (BF/HR/S-TEM), time-of-flight elastic recoil detection analysis (TOF-ERDA), time-of-flight secondary ion mass spectrometry (TOF-SIMS), ultraviolet-visible spectroscopy (UV-Vis), soft X-ray absorption spectroscopy (SXAS), grazing-incidence X-ray diffraction (GI-XRD), X-ray photoelectron spectroscopy (XPS), X-ray fluorescence (XRF), and X-ray reflectometry (XRR).

Atomic layer deposition enables the coating of electrode materials even in aggregate/powder form, prior to binding and preparing the active material into an electrode; this allows for increased leveraging of the functionality of the thin film material.^13^ However, the coating then needs to be electrically conductive to facilitate charge transfer within the electrode and thus its basic functioning. If the deposition is done onto a bulk or pre-bound and prepared electrode, an electric insulator will also work and is often preferrable, as this lets it double as a solid-state electrolyte (SSE). In both cases, robust ionic conductivity is desired.

## Protective barrier coatings

There is already a variety of ALD processes utilized in the deposition of prospective electrode coating materials. In this section we discuss inorganic ALD materials and processes that were targeted as protective coatings in battery systems, but which do not specifically mimic the spontaneously forming SEIs. Selected examples of these materials and processes are listed in Table 2, and those containing alkali metals in particular are listed in Table 3.

**Table 2:** Examples of ALD and ALD/MLD processes for protective electrode coatings, with notes on the electrode preparation schemes and characterization methods employed

Material	Precursor 1	Precursor 2+	Substrate	Electrode preparation	Characterization	Ref.
Al_2_O_3_	TMA	H_2_O	Li metal	Punch-out disks	Cyc, EIS, QCM, SEM, TEM, XPS, XRF	45
			Hard carbon	Slurry applied on Cu foil and dried	Cyc, EIS, SEM, TEM, XPS	66
			Na metal	Cut slices from metal cubes	Cyc, EIS, SEM, XPS	67
			TiO_2_ nanotubes	Ti foil anodized in a cell and annealed to anatase crystalline phase	CA, Cyc, EIS, FE-SEM	68
			Na metal	Metal stick pressed into foil	Cyc, QCM, RBS, SEM, XPS	69
Al_2_O_3_ TiO_2_	TMA TiCl_4_	H_2_O	Si micropillars	Lithograph and ICP etching of bulk Si(100)	Cyc (initial lithiation only), FIB, FE-SEM, (BF)/(HR)TEM	48
TiO_2_	TTIP	H_2_O	Li metal	Li foil, polished	Cyc, EDX, EIS, SEM, XPS	70
AlO_*x*_N_*y*_	TMA	N_2_/H_2_ gases	Si metal	Si nanoparticles in rubber, dried	CV, Cyc, EIS, HRTEM, SEM, XPS	71

**Table 3:** Examples of alkali-metal-containing ALD and ALD/MLD processes tested as artificial SEIs, deposited onto electrodes after they were prepared as described herein. Characterizations performed on these depositions are also detailed

Material	Precursor 1	Precursor 2+	Substrate	Substrate structure	Characterization	Ref.
LiP_*x*_O_*y*_N_*z*_	Li–HMDS	DEPA	Pt	Pt film deposited on Si	CP, CV, EIS, SEM	51
	LiO^*t*^Bu	H_2_O, PO(OMe)_3_, N_2_-plasma	(Various)	(Various)	(Various)	72–75
		H_2_O, PO(OMe)_3_, N_2_	Li Li_10_GeP_2_S_12_	Punch-out disks Powder pressed into pellets	CV, Cyc, EIS	76
		NH_3_, P(NMe_2_)_3_, O_2_	Au, glass	Interdigitated Au on glass	EIS, SEM, (S)TEM, XPS	50
LiP_3_O_4_	LiO^*t*^Bu/Li–HMDS	PO(OMe)_3_	Glass	Soda-lime glass squares	ERDA, HTXRD	77
	LiO^*t*^Bu	PO(OMe)_3_, H_2_O	Steel	Premade stainless steel	CV, EIS	72
LiF	LiO^*t*^Bu	HF	Li Pt	Punch-out disks Interdigitated Pt electrode	Cyc, EIS, SEM	78
		TiF_4_ HFAC	LiMn_1.5_Ni_0.5_O_4_	Powder	CV, Cyc, EIS, HRSEM, ICP, TEM, TOF-SIMS, XPS	49
NaP_*x*_O_*y*_N_*z*_	NaO^*t*^Bu	DEPA	Pt	Pt film with Ti adhesion on Si_3_N_4_-passivated Si(100)	EIS	79
Li_3_BO_3_–Li_2_CO_3_	LiO^*t*^Bu	O_3_, B(O^*i*^Pr)_3_	Graphite	Laser-patterned, postcalendered	Cyc, EIS, (FIB-)SEM, XPS	80
LiAl_*x*_Zn_*y*_O_*z*_	LiO^*t*^Bu	TMA, DEZ, H_2_O	LiNiO_2_	Powder; calcined Ni(OH)_2_ + LiOH	Raman, SEM, STEM, SXAS, XPS, XRD	81
Li–HQ	LiO^*t*^Bu	HQ	Li	Li foil	AFM, Cyc, EIS, FE-SEM, NI, XPS, XRD	82
				Li chip	Cyc, EDX, EIS, SEM, XPS, XRD	83
LiO_2_–Li–HQ	LiO^*t*^Bu	HQ, H_2_O	Si electrode	Nano-Si, carbon and binder	AFM, CV, Cyc, EIS, GITT, NI, TEM, TOF-SIMS, XPS, XRD	84
Li–GL	LiO^*t*^Bu	GL	Li	Punch-out disks	AFM, Cyc, EDX, EIS, SEM, XRD	85
			Li Au	Li anode Interdigitated Au electrode	Cyc, EIS, (FIB-)SEM, (S)TEM, XPS	54
Li–TEA	LiO^*t*^Bu	TEA	Li	Punch-out disks	Cyc, EDX, EIS, SEM, XPS, XRD	55

**Aluminum oxide (Al**_**2**_ **O**_**3**_ **)** is a commonly deposited ALD material for various barrier applications; accordingly, ALD–Al_2_O_3_ thin films have been employed as protective coatings in different electrochemical applications. It is also by far the most studied among ALD-made electrode coatings. Al_2_O_3_ is usually deposited using trimethylaluminum (Al(CH_3_)_3_, TMA) and water as the precursors, and sometimes ozone (O_3_) or O_2_ plasma in place of the latter. Both TMA and water are liquids with vapor pressures at the 10–20 mbar range at room temperature, allowing for straightforward precursor evaporation and making the deposition process simple and compatible with many ALD reactor types. Only the substrate needs heating (to 80–200 °C), and short pulses (of ≤1 s) are often sufficient for film growth at a GPC of ∼1 Å.^45,66–69^ As this ALD process is well-developed and easily achieves highly conformal films thanks to the volatile and reactive precursors,^31^ it is particularly useful for coating challenging 3D surfaces. Indeed, ALD–Al_2_O_3_ has been used to passivate both cathode– and anode–electrolyte interfaces in LIBs, even on high-aspect-ratio surfaces.^69^ Al_2_O_3_ coatings can improve the surface wettability with the electrolyte, and thereby evenly distributing the ion flux into the material through the interfaces. This facilitates the development of a thinner, uniform natural SEI and reduces electrolyte consumption, leading to significantly improved capacity retention during cycling.^45,67,68^ The same has also been successfully applied to sodium-ion batteries; a <3 nm Al_2_O_3_ coating endowed the battery with an extended lifetime.^67^

**Titanium oxide (TiO**_**2**_ **)** is another simple ALD material that has been studied as a protective electrode coating. It has been deposited using either titanium(iv) isopropoxide (TTIP) or TiCl_4_ as the titanium source, and H_2_O as the oxygen source. In one such process, TTIP and water were added in short pulses (∼1 s) to deposit films at 150 °C. The resulting TiO_2_ films were amorphous, although when coated onto Li-metal anodes, they reacted with Li metal to form crystalline Li_*x*_TiO_2_. This chemical/structural transformation improves the ionic conductivity of the thin films, and was reported to improve the lifetime of the anode 8-fold at a 1 mA h cm^−2^ current density.^70^

**Fig. 5:**
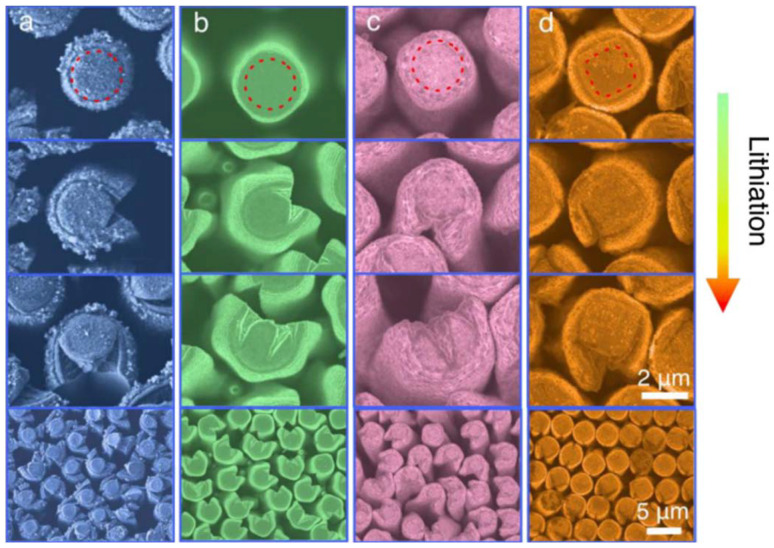
Cross-sections of Si micropillars over the course of lithiation: (a) bare, (b) Al_2_O_3_- and (c) TiO_2_-coated circular pillars; and (d) Ti_2_O_3_-coated square pillars. The lithiation of Si leads to large volume expansion, which is evident from the visibly fractured SEI (a) and ALD coatings (b–d), with a square pillar shape (d) appearing to mitigate this to noticeable degree. Reprinted from *Enhanced lithiation and fracture behavior of silicon mesoscale pillars via atomic layer coatings and geometry design*,^48^ © 2014, with permission from Elsevier.

In one of the early studies, both aluminum and titanium oxides were deposited on Si micropillars by ALD to compare their ability to mitigate natural SEI formation as well as accommodate the significant volume-change inherent to Si upon lithiation.^48^ It was found that the ALD coatings resulted in a significantly thinner spontaneous SEI (<0.15 μm) than on bare Si pillars (0.5–0.8 μm). Interestingly, the coated samples exhibited strong anisotropic volume expansion (a characteristic of Si nanowires and -pillars); this was ascribed to SEI formation decisively affecting lithium transport. The shape of the micropillars also influenced the coating durability, wherein square (by cross section) micropillars retained a more intact coating than circular ones (Fig. 5).

The Al_2_O_3_ and TiO_2_ ALD processes typically yield highly conformal coatings,^31,86^ but these coatings suffer from somewhat low ionic conductivity, which is exacerbated more the thicker the films are. Charge carrier ion consumption *via* formation of a combined tertiary structure can also be an issue. Thus, it becomes useful to explore outside the domain of simple/binary metal oxide films.

One promising class of ionic conductors (and electronic resistors) deposited *via* ALD are the alkali phosphorus oxynitrides, LiP_*x*_O_*y*_N_*z*_ and NaP_*x*_O_*y*_N_*z*_ (LiPON and NaPON). Depending on the ALD process parameters, different stoichiometries can be achieved for these materials. Although the simpler phosphate (LiP_3_O_4_) has been deposited and deemed a reasonable ionic conductor,^20,72,77^ a suitably high nitrogen content in the form of P–N bonds is seen to further benefit ionic conductivity.^50,95^ Multiple ALD processes exist to deposit the material with these bonds present, each with their own benefits and drawbacks. For example, the choice of lithium precursor can impact the conformality and depth of LiPON growth on challenging 3D surfaces.^96^

For LiPON, the lithium precursors LiO^*t*^Bu and Li–HMDS have been used at 100–180 °C and 60 °C, respectively. The other elements, P, O and N, have been delivered into the LiPON film by one of two ways: (i) with diethyl phosphoramidate (DEPA, 85–115 °C) in a binary process,^43,95^ or (ii) in quaternary processes using separate sources for O (H_2_O or O_2_), N (^P^N_2_ or NH_3_) and P (PO(OMe)_3_ or P(NMe_2_)_3_).^50,72–74^ Ionic conductivity values ranging from 10^−8^ to 10^−6^ S cm^−1^ have been recorded for LiPON films, depending on the elemental composition and degree of crystallinity, while electrical conductivity has been below 10^−10^ S cm^−1^.^95^ The structure and properties of these materials bestow them with multiple uses, including as electrode coatings.

**Fig. 6:**
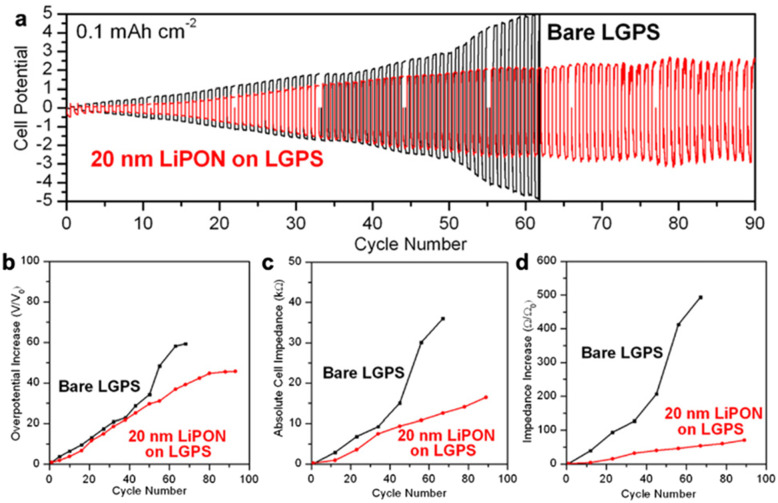
Electrochemical performance of bare and 20 nm LiPON ALD-coated Li_10_GeP_2_S_12_ (LGPS) assembled in Li/LGPS/Li cells. (a) Galvanostatic cycling (current density 0.1 mA cm^−2^; charge capacity 0.1 mA h cm^−2^); (b) increase in overpotential, (c) absolute impedance and (d) impedance increase of the cells during cycling. Reproduced from *Differentiating chemical and electrochemical degradation of lithium germanium thiophosphate and the role of atomic layer deposited protection layers*,^76^ under CC BY-NC 3.0.

Thin films of LiPON have been observed to mitigate the loss of Li-ions on a coated electrode, which helped significantly boost the lifetime of the electrode, though a prerequisite was the fabrication of properly formed films with no defects;^97^ ALD has been an effective tool for depositing films befitting these criteria.^72,95^ Moreover, ionically conducting ALD–LiPON can be used to passivate interfaces between Li electrodes and a superionic conductor Li_10_GeP_2_S_12_, where high-impedance layers would otherwise form due to Li diffusion into the Li_10_GeP_2_S_12_ phase.^76^ Cycling tests performed on the LiPON-coated Li_10_GeP_2_S_12_ showed significantly improved electrochemical performance, including more stable cell potential, and a lower increase in cell overpotential and impedance over the course of testing (Fig. 6), even though the degradation of Li_10_GeP_2_S_12_ could not be fully prevented.

NaPON has been deposited using a process akin to the binary LiPON process, with NaO^*t*^Bu (at 155 °C) as the Na precursor and DEPA (115 °C) as the second reactant. Ionic conductivity for the NaPON film was measured within the range of LiPON films, at 10^−7^ S cm^−1^.^79^ Further studies on the electrical and electrochemical properties of NaPON electrode coatings remain yet to be conducted.

Alkali fluorides, LiF and NaF, are typically found in spontaneously forming SEIs in alkali-ion batteries due to the commonly used fluorine-containing electrolytes, *e.g.*, LiPF_6_. As alkali fluorides are relatively straightforward to prepare by ALD, this has made them popular candidates for electrode coatings. Although they do not possess a meaningful ionic conductivity, they are highly stable and insoluble even with liquid electrolytes.^5,78^ LiF and NaF have been deposited pairing Li- or NaO^*t*^Bu at 130–230 °C with TiF_4_ (120–130 °C), HF (kept at RT) or HFAC (hexafluoroacetylacetone, kept at RT) as the fluorine precursor; however, in the last case, a hybrid LiF–CF_*x*_ structure instead of LiF emerged.^49,78,98–100^ Both fluorides are crystalline as-deposited.^49,100^ Electrodes coated with LiF had their charge–discharge cycling stability significantly improved compared to the bare electrodes, and the cells showed >99% (coulombic) efficiency.^100^ LiF has also been deposited in consecutive cycles with AlF_3_ at a 1 : 1 ratio to form LiAlF_4_. These films were measured to have an ionic conductivity of ∼3.5 × 10^−8^ S cm^−1^, several times higher than that reported for the ALD–LiF films, while also showing the beneficial stable properties.^99^

**Fig. 7:**
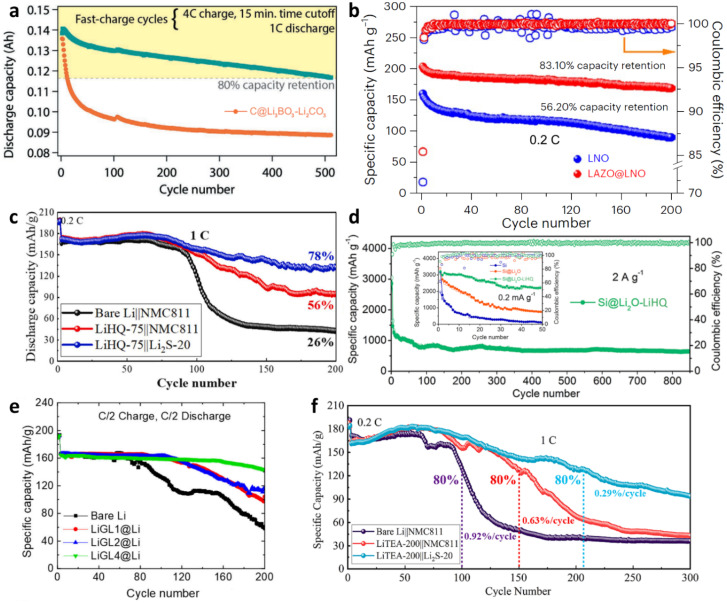
The effect of ALD and ALD/MLD films on the capacity retention of various cells during galvanostatic cycling: (a) Li_3_BO_3_–Li_2_CO_3_ on graphite,^80^ (b) LiAl_*x*_Zn_*y*_O_*z*_ on LiNiO_2_,^81^ (c) Li–HQ on Li metal,^83^ (d) Li_2_O–Li–HQ on Si,^84^ (e) Li–GL on Li metal,^54^ (f) Li–TEA on Li metal.^55^ The abbreviations of the organic moieties are listed in Table 3. (a) Reproduced from *Enabling 4C Fast Charging of Lithium-Ion Batteries by Coating Graphite with a Solid-State Electrolyte*.^80^ © 2021 Wiley-VCH GmbH. Reproduced with permission. (b) Reproduced from *High-energy all-solid-state lithium batteries enabled by Co-free LiNiO_2_ cathodes with robust outside-in structures*,^81^Springer Nature. Reproduced with permission from SNCSC. (c) Reprinted from *A novel polymeric lithicone coating for superior lithium metal anodes*,^83^ © 2024, with permission from Elsevier. (d) Reprinted (adapted) with permission from *Atomic/Molecular Layer-Deposited Laminated Li_2_O*–*Lithicone Interfaces Enabling High-Performance Silicon Anodes*.^84^ © 2023 American Chemical Society. (e) Reproduced from *Lithicone-Protected Lithium Metal Anodes for Lithium Metal Batteries with Nickel-Rich Cathode Materials*,^54^ under CC BY 4.0. (f) Reprinted from *Tackling issues of lithium metal anodes with a novel polymeric lithicone coating*,^55^ © 2023, with permission from Elsevier.

While the materials discussed thus far represent more commonly deposited categories, many others have been fabricated. A collection of capacity retention tests for these is shown in Fig. 7. While the testing parameters are frequently not comparable between studies, the broader conclusion is that the coatings can provide significant battery longevity benefits, especially for unstable high-capacity electrode materials such as Li metal and Si. While uncoated electrodes tended to suffer from large capacity drops within the first 200 cycles, the coated electrodes had no such issue and instead slowly lost capacity over many hundreds more cycles.^54,55,80,81,83,84^ In the longest test run in terms of cycles (Fig. 7d), the capacity remained stable and higher than for conventional electrode materials for the entire almost-1000 cycles.^84^ Among inorganic thin films, a mix of lithium borate and carbonate almost eliminated the initial steep capacity drop of a graphite electrode, instead declining slowly but steadily by 20% of its capacity over 500 cycles at a fast 4C rate (Fig. 7a; *n*C = 1/*n* hours to fully charge the battery).^80^ A LiNiO_2_ electrode coated with LiAl_*x*_Zn_*y*_O_*z*_ had notably improved capacity already on its first cycle at 0.2C, and declined more slowly than an uncoated one especially after 100 cycles (Fig. 7b).^81^

## Prospective ASEI materials by ALD/MLD

Several ALD/MLD metal–organic thin-film materials have been deposited to mimic the composition and structure of spontaneously forming SEI layers (Table 4). In state-of-the-art alkali-ion batteries, the naturally occurring SEI layer typically contains decomposition products of the organic electrolyte solvent, *i.e.*, organic carbonates.

**Table 4:** Potential new ASEI materials made by ALD and MLD

Material	Precursor 1	Precursor 2+	Substrate	Characterization	Ref.
Al–EG	TMA	EG	(Various)	(Various)	69 and 87–89
Zn–EG	DEZ	EG	Si electrode	CV, EIS, TOF-SIMS, XPS, XRD	90
Zn–ED–PDIC	DEZ	ED, PDIC	Li foil	CT, Cyc, TOF-SIMS, XPS	91
Ti–HQ	TiCl_4_	HQ	Si-nanoparticle, Ti_3_C_2_T_*x*_, CNT composite electrode	AFM, CV, Cyc, EIS, EDS, Raman, SEM, TEM, XPS	92
Zn–HQ	DEZ	HQ	Cu nanowire	Cyc, ECSA, EDX, SEM, TEM, XPS, XRD	46
Ti–DCA	TDMAT	(Various DCAs)	PVD TiO_2_, TiN, LiMn_2_O_4_	CV, Cyc, XPS	10
Li–EG(–CO_2_)	Li–HMDS	EG, (CO_2_)	Si	AFM, FTIR, SEM, XRR	53
Li–EG	LiO^*t*^Bu	EG	Si; Pt electrode	AFM, CA, EIS, FTIR, SE, SEM, XPS, XRD	93
Li–HQ	LiO^*t*^Bu	HQ	Si; Li electrode	AFM, Cyc, EIS, FE-SEM, FTIR, NI, XPS	82
Li_2_O–Li–HQ	LiO^*t*^Bu	H_2_O, HQ	Si electrode	AFM, EDS, FTIR, GIXRD, NI, TEM, TOF-SIMS, XPS	84
VC	APS	VC	Treated graphite powder	EDS, BF-STEM, TEM, XPS	94
PU	ED	PDIC	Li foil	Cyc, EIS, SEM, TOF-SIMS, XPS	52

Inclusion of the organic moieties in the film structure necessitates the use of MLD. While the natural SEI has been observed to be an irregular mosaic of inorganic and organic constituents such as Li_2_O, LiF, Li_2_CO_3_ and Li ethylene carbonate,^22^ an ALD/MLD grown film possesses a more regular thin-film structure. In fact, the aim is that the more regular structure by ALD/MLD would have fewer chemical and mechanical weak points and would thus deteriorate more slowly than the naturally forming and more defected SEI layer, thus also reducing the occurrence of charge-carrier-consuming side reactions and enhancing the longevity of the battery.^101^ Naturally, the ALD/MLD grown ASEI layer should be stable in the electrochemical environment of the battery, like the spontaneous SEI.

Commonly employed ALD and MLD precursors – TMA and EG, respectively – have been combined to deposit aluminum–ethylene-glycol (Al–EG) as a coating for Na-metal electrodes. The volatility of these liquid (at RT) precursors enabled a deposition temperature as low as 85 °C, and 60 nm thick Al–EG films were shown to mitigate the dendrite formation – a typical problem for alkali-metal electrodes. This helped allow more stable operation and a longer lifetime of the battery, in comparison with batteries having either the bare or the ALD–Al_2_O_3_ coated Na electrode. The advantages were partly attributed to the increased flexibility and mechanical durability of Al–EG during Na plating and stripping.^69^

In addition to the simple EG, more complex MLD precursors have been studied to investigate the effects of the organic component on the material properties. Hydroquinone can be considered an analogue to EG, but with a benzene ring in place of the single C–C moiety. In one such study, diethyl zinc was combined with hydroquinone to deposit Zn–HQ, where the nucleophilic behavior of HQ was utilized to decompose an electrolyte component to incorporate LiF into the SEI.^46^ Additionally, aliphatic dicarboxylic acids with varying chain lengths (oxalic, malonic, succinic, glutaric and 3,6-dioxaoctanedioic acids) have been tested in tandem with Ti precursor Tetrakis(dimethylamino)titanium(iv), deposited on top of various cathode materials. Of these, Ti–oxalic-acid showed saturative ALD/MLD-growth, while the others exhibited a CVD-like component with the Ti-precursor pulse. The resulting Ti–carboxylate-coated cathodes were extensively characterized by CV, whereupon wider stable potential windows during delithiation were seen than for a comparative ALD/MLD material, Ti–glycerol.^10^

Closer approximations of naturally occurring SEIs in alkali-ion batteries can be achieved by including the charge-carrying alkali metal in the ALD/MLD ASEI film structure during deposition. This is achieved by using an alkali metal precursor – such as LiO^*t*^Bu, Li–HMDS or NaO^*t*^Bu – in the ALD/MLD process, instead of the more common metal precursors containing Al, Ti or Zn. A simple lithium ethylene glycoxide (Li–EG) film has been deposited in two studies to date, with Li–HMDS^53^ or LiO^*t*^Bu^93^ as the Li precursor. Li–HMDS has a significantly lower vaporization temperature and is usable at just 55 °C, which combined with EG at 30 °C allowed for a low deposition temperature of 80 °C.^53^ In the latter study, electrochemical properties of the films were examined as well, with an ionic conductivity recorded within 3.6–5 × 10^−8^ S cm^−1^ near RT at a 0.6 eV activation energy.^93^

Li–HQ has been observed improving the cycling behavior of both Li metal^83^ and Si^84^ electrodes. The Li-metal electrodes were cycled at 100 and 200 mA g^−1^ rates in cells with a bare and ALD–Li_2_S-coated Li(Ni_0.8_Mn_0.1_Co_0.1_)O_2_ cathodes. The cells with both Li_2_S- and Li–HQ-coated electrodes fared the best, especially when tested at the faster rate, where the cell with bare Li metal suffered dramatic capacity loss at ∼100 cycles, while the cell with both electrodes coated slowly declined to ∼80% capacity over 200 cycles (Fig. 7c). Modest improvement was seen also at the slower rate, though significant capacity decline happened over 500 cycles.^83^ Silicon electrodes were coated with a Li_2_O–Li–HQ superstructure and at a slow rate (0.2 mA g^−1^) could preserve a capacity of over 2000 mA h g^−1^ for 50 cycles, and at a faster rate (2 mA g^−1^) degraded quickly to ∼800 mA h g^−1^, but remained notably stable there for over 800 cycles (Fig. 7d).^84^

Glycerol is also analogous to EG, but with an additional hydroxymethyl (–CH_2_–OH) group. Lithium metal electrodes have been coated with Li–GL, with thicker coating (400 ALD/MLD cycles) yielding better stability over 200 cycles at 0.5C than thinner (200 and 100 cycles) depositions (Fig. 7e).^54^ A somewhat different organic molecule – triethanolamine (TEA) – was trialed with a similar setup as in the Li–HQ study, in cells with Li_2_S-coated and bare Li(Ni_0.8_Mn_0.1_Co_0.1_)O_2_ cathodes, exhibiting similar stability with both electrodes coated (with 80% capacity remaining after 200 cycles at 1C; Fig. 7f).^55^

**Fig. 8:**
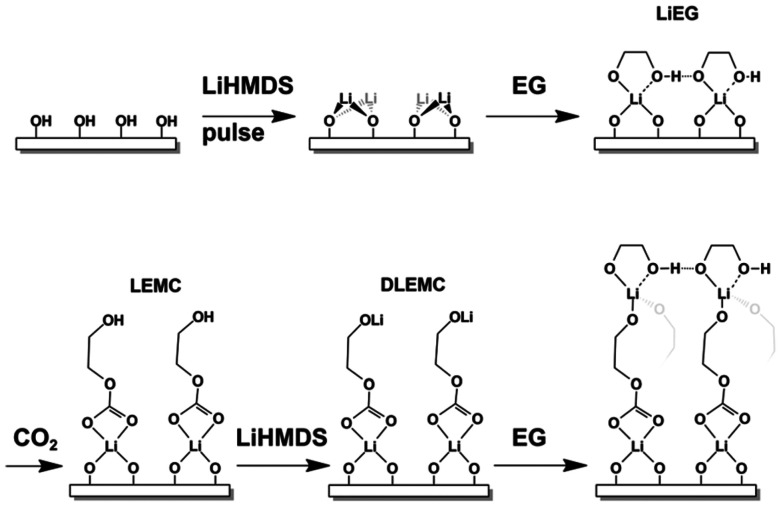
A possible growth mechanism for Li–EG–CO_2_ thin films, where CO_2_ is inserted between the previous two materials (Li and EG). Reproduced from *CO_2_-based atomic/molecular layer deposition of lithium ethylene carbonate thin films*,^53^ under CC BY-NC 3.0.

In post-mortem analyses of Li-ion batteries employing the common carbonate-based liquid electrolyte, lithium ethylene carbonates have been found as part of the SEI structure. Thus, to create a closer analogue to natural SEI films, CO_2_ can be added to the Li–EG process as a third precursor. The CO_2_ inserts itself into the Li–O moiety of the Li–EG structure (Fig. 8), forming lithium ethylene mono carbonate (Li–EG–CO_2_). This is possible without adverse effects even if only pulsing CO_2_ every few ALD cycles as opposed to every cycle.^53^ Though detailed analyses are still to be performed, the ALD/MLD Li–EG–CO_2_ films were already successfully deposited onto thin-film Li-terephthalate batteries without impeding their initial cycling performance.^102^

In addition to hybrid ALD/MLD, purely organic MLD films have also been tested. One process described as MLD involved long single-layer depositions of first (3-aminopropyl)triethoxysilane (APS), and then the commonly used SEI-former, vinylene carbonate (VC), onto graphite.^94^ This contrasts with most other ALD and MLD processes and their repeated pulses and multiple atomic or molecular layers, and it highlights the diversity of the methods, both in terms of definition and applications. A different, more typical MLD process involved alternating depositions of ethylene diamine (ED) and 1,4-phenylene diisocyanate (PDIC) to create polyurea (PU) thin films that conferred a significant longevity advantage to the coated Li-metal anodes.^52^

## Conclusions and outlook

Interfacial stability remains a key obstacle with Li- and Na-based batteries, slowing down both the improvement of conventional battery cells and the introduction of novel high-capacity electrode materials. Among the methods employed to stabilize the electrode–electrolyte interface, pre-fabrication of an artificial solid electrolyte interphase coating using atomic and molecular layer deposition – techniques with unparalleled accuracy and tailorability – is promising both in theory and in results obtained thus far. Improvements have been seen both on the commonly used graphite, and particularly on prospective high-capacity but low-lifetime materials such as Li- and Si–metal electrodes, whose charge–discharge performance was in many test cases transformed from dramatic capacity drops over the first 50–200 cycles to sustained performance for 200–800+ cycles.

Owing to the benefits of ALD, coating is possible not only on already assembled electrodes, but also on electrode aggregates/powders prior to binding into an electrode, thus enabling more thorough modifying of the properties of the electrode material. Solutions for the ALD-coating of battery materials have already been built and used commercially on an industrial scale, and it is easy to expect this development to continue.

While common coating materials like Al_2_O_3_ have remained the default option used for interface modification by ALD, the versatility of the technique has increasingly been used to already include the charge carrier ions (Li, Na) in the deposited ASEI layer. Various materials with unique combinations of properties have been fabricated using these techniques, and particularly the combined ALD/MLD approach enables the creation of ASEIs with desirable ionic conductivity, mechanical flexibility and electrochemical stability. Future material experiments that capture the stability of the natural SEI structure while deviating from them enough to improve these properties should prove particularly interesting.

While the number of studies exploring ALD/MLD ASEIs is still comparatively small, the subject has picked up progressively more interest in the last several years. So far, ALD/MLD coatings have yielded significant improvements in the longevity of high-capacity battery materials, with the vastly increased number of stable charge–discharge cycles approaching commercially useful stability. Still, due to the vast possibilities in material combinations alone, more research is needed to home in on the ideal material for each type of battery cell. Given the relative expense and difficulty of employing these techniques at scale, particularly with less common ALD and ALD/MLD materials, it is imperative to confirm these results and further improve on them to capture commercial interest.

## Author contributions

The manuscript was written through contributions of all authors.

## Conflicts of interest

There are no conflicts of interest to declare.

## Data Availability

No primary research results, software or code have been included and no new data were generated or analysed as part of this review.
